# Supplementary material for: Dietary Berberine and Ellagic Acid Supplementation Improve Growth Performance and Intestinal Damage by Regulating the Structural Function of Gut Microbiota and SCFAs in Weaned Piglets
Source: Microorganisms. 2023 May 10;11(5):1254. doi: 10.3390/microorganisms11051254 (PMC10220671; doi:10.3390/microorganisms11051254)
Supplement: Supplementary file 1 [file microorganisms-11-01254-s001.zip › microorganisms-2306748-supplementary.pdf]

Table S1. Primer sequences for RT-qPCR

Target gene	Forward and reverse	Primer sequence (5′-3′)
Occludin	Forward primer	GCTGGAGGAAGACTGGAT
	Reverse primer	ATCCGCAGATCCCTTAAC
Claudin-1	Forward primer	TACTTTCCTGCTCCTGTC
	Reverse primer	AAGGCGTTAATGTCAATC
ZO-1	Forward primer	ACCCACCAAACCCACCAA
	Reverse primer	CCATCTCTTGCTGCCAAACTATC
β-actin	Forward primer	TCTGGCACCACACCTTCT
	Reverse primer	TGATCTGGGTCATCTTCTCAC
